# Research and Remedies

**Published:** 1913-05-24

**Authors:** 


### RESEARCH AND REMEDIES.

### Effects of Digitalis on Arterial Pressure.

As the result of work done by Josué and Godlewski, and reported to the Société Médicale des Hôpitaux, it would appear that digitalis does not increase the arterial tension, except in cases where there is cardiac insufficiency accompanied by hypotension. In patients with normal tension, and in arterio-sclerotics with a raised tension, digitalis does not elevate the blood-pressure. Thus the drug can be given without risk to the latter if it is desirable to regulate the function of the heart. These conclusions, which are quite at variance with established ideas, have been arrived at as the result of numerous very careful measurements. Another fact which the authors have emphasised (it has long been acknowledged and accepted by clinicians from bedside experience) is that arterial pressure undergoes considerable alterations at various times in the day in the same person. Thus a single observation daily is not of much use in observing arterial pressure. This last should be taken two or three times in the morning and once in the afternoon, preferably for several days consecutively.

### Cæsarean Section for Placenta Prævia.

A good many obstetricians have considered Cæsarean section for complete placenta prævia in favourable cases, and a few have put the idea into practice. Foulkrod, in the *American Journal of Obstetrics and Gynæcology*, goes further still. He would treat in this way any case of complete or partial placenta prævia in preference to vaginal interference with dilators, bags, forceps, and so on. When a practised operator and reasonably favourable conditions for an aseptic environment can be secured, Foulkrod's teaching is probably right, and in a few years may become generally accepted. The arguments which he presents in favour of Cæsarean section in this condition are as follows: The placental site is left absolutely undisturbed; hæmorrhage and chances of infection are therefore much diminished. The shock of operation is no greater—indeed, he thinks, probably less. The child has an infinitely better chance of life. The mother's chance is as good as by the older methods, and she is left in a better physical condition. In unhandled cases the possibility of sepsis is much reduced.

